# Safer Pediatric Sedations: Simulation Checklists to Improve Knowledge, Attitudes, and Skills in Emergency Medicine Residents

**DOI:** 10.7759/cureus.70516

**Published:** 2024-09-30

**Authors:** Lawrence Lau, Ronald V Hall, Dimitrios Papanagnou, Kory London

**Affiliations:** 1 Emergency Medicine, Kaiser Permanente Medical Group, San Leandro, USA; 2 Department of Emergency Medicine, Thomas Jefferson University, Philadelphia, USA

**Keywords:** checklist development, deep sedation, pediatric emergency and acute care, procedural sedation and analgesia, quality improvement and patient safety, simulation in medical education

## Abstract

Background

Pediatric sedation is a low-frequency, high-stakes procedure. This study aimed to train emergency medicine (EM) residents in pediatric procedural sedation through a sedation checklist, enhancing patient safety.

Methods

EM residents completed a pre-test and a survey on their knowledge and experiences with sedation protocols. Residents were subdivided into four groups: two control groups underwent a pediatric sedation simulation without the aid of a procedural checklist, while two intervention groups were given the procedural checklist to guide their management of the procedure. Following the simulations, a simulation faculty member reviewed sedation management and safety with residents for all groups and answered questions. An improvement analysis was performed via a post-intervention examination among all residents.

Results

Residents in the intervention group demonstrated an improvement in obtaining more critical actions during the simulation (intervention group critical actions 14, 13 vs non-intervention critical actions 10, 12) and confidence with the procedure (via a Likert scale survey across multiple arenas of pediatric sedation), with only moderately increased scores on the post-test examination (pre-simulation score of 6.28±2.14; post-simulation score of 6.75±1.88).

Conclusion

The data suggest that a checklist, combined with dedicated training through simulation, improves knowledge, confidence, and skill with regard to pediatric sedations. Further study is required to examine the longitudinal impact of our program on resident performance and patient outcomes.

## Introduction

Pediatric procedural sedation is a high-stakes procedure demanding frequent review of technique, complications, and training in management during emergent situations. Its utility in providing analgesia, anxiolysis, and amnesia to children undergoing invasive procedures, diagnostic imaging, or painful orthopedic reductions makes this a crucial topic domain in emergency medicine (EM) [1]. In 2006, a large multicenter study by the Pediatric Sedation Research Consortium reviewed pediatric sedations performed across 26 institutions. In 30,000 pediatric sedations performed outside of the operating room, including those performed by anesthesia, pediatric specialists, emergency department (ED) physicians, and other health providers, the authors reported no incidence of death; however, complications (i.e., the development of stridor, laryngospasm, wheezing, apnea) were noted in one out of 400 cases. One in 200 sedations required intervening airway management techniques such as bag-valve-mask ventilation, placement of an oral airway, or definitive intubation [2]. The probability of adverse reactions increased significantly with an upper respiratory tract infection, which are quite common in pediatric patients [3-5]. Advances in airway management, namely end-tidal carbon dioxide (ETCO_2_), have been shown to improve the detection of apnea and hypoxic events prior to oxygen desaturation. This allows for quick airway management, including physical stimulation, airway manipulation, nasal or oral airway, oxygen supplementation, and positive pressure ventilation for the mitigation of life-threatening situations [6,7]. For an inexperienced operator, failure to recognize these impending complications allows for degeneration into life-threatening situations, if not managed appropriately. Checklists have also become increasingly utilized in the healthcare setting, with multiple large trials and systematic reviews showing evidence of improved outcomes and lower rates of adverse events [8-11].

In EDs where the pediatric volume may be low, the review of safety checklists and procedural skills is imperative to ensure operator competency and promote shared mental models among staff during sedations. While there are educational considerations in EM residency training to provide for the acquisition of skills required during these sedations (i.e., resident lectures, informal and incidental learning in the workplace, small group workshops), the training models that prevail are suboptimal. Critical steps may be overlooked, and without deliberate practice, both procedural skills and procedural knowledge can decay with time.

Simulation training presents a ripe opportunity to review procedural checklists, support the development of a shared mental model with the entire clinical team, deliberately practice procedural skills under the supervision of a senior resident or faculty member through formative feedback, and support the development of muscle memory for procedural skills when they are needed most. Best of all, it is congruent with patient safety efforts.

The Accreditation Council for Graduate Medical Education (ACGME) already mandates resident competency for performing high-stakes procedures, along with managing and troubleshooting potentially adverse complications, prior to graduation [12]. Pertinent resident milestones include the following:

General Approach to Procedures (PC9): “Performs indicated procedure on any patients with challenging features . . . takes steps to avoid potential complications, and recognizes the outcome and/or complications resulting from the procedure” (Level 4)

Airway Management (PC10): “Performs airway management in any circumstance, taking steps to avoid potential complications, and recognizes the outcome and/or complications resulting from the procedure” (Level 4)

Procedural Sedation (PC11): “Performs procedural sedation providing effective sedation with the least risk of complications and minimal recovery time through selective dosing, route, and choice of medications” (Level 4)

The overarching goal of our project was to improve the competency of EM residents in performing pediatric procedural sedations in the ED and managing potential complications that may arise. The specific aims of the study were to 1) to identify the critical steps/actions for pediatric procedural sedations, practiced by EM residents prior to sedation, 2) to develop an interprofessional checklist for pediatric procedural training and practice, and 3) to measure the impact of a pediatric procedural program on resident behaviors, attitudes, and procedural success.

The importance of this study lies in its ability to both advance simulation training in EM and impact patient safety. Through this intersection of simulation technology and evidence-based pedagogy, we aimed to demonstrate that a robust bundle, inclusive of a checklist with critical actions to be practiced, can successfully prepare residents for success with sedations.

## Materials and methods

This study is a descriptive analysis of a real-world educational intervention provided to residents in the Thomas Jefferson University Emergency Department. Content experts in pediatric airway management were recruited from the department and university to capture the critical steps and actions needed to successfully prepare for, perform, and manage a safe pediatric sedation, as well as handle potential adverse events such as medications and airway techniques. Content experts with simulation background were chosen from the specialties of EM, pediatrics, anesthesia, as well as members of our patient safety department. In addition, the Jefferson Center for Interprofessional Education (JCIPE), the nation’s only center for advancing interprofessional education (IPE) and collaborative practice in the health sciences, was consulted to ensure that the checklist includes core IPE considerations. A modified Delphi analysis was performed, with serial surveys to determine the list of critical supplies, knowledge, and actions, and formulated into a comprehensive pediatric sedation checklist shown in Appendix Figures 1-5). When consensus had been reached, the instruments were piloted with two resident volunteers to assess construct and content validity with minor semantic revisions providing the final document. This study was assessed by the Thomas Jefferson University Institutional Review Board and approved using standard informed consent.

A total of 38 residents in the EM Residency Program at Thomas Jefferson University (i.e., post-graduate year [PGY] 1, 2, and 3) were provided a pre-test on their knowledge of pediatric ED sedations (i.e., indications, medications, technique, rescue interventions), as well as a survey on their attitudes towards the procedure (i.e., experience level with pediatric sedations, number of sedations performed, and confidence level with sedations, scored on a 5-point Likert scale). The pre- and post-exposure testing had 100% response rates. The pre-test and the survey are included in Appendix Figures 6-8), respectively. Data were collected through an online Google survey link.

Following data collection over the course of one month, PGY-1, PGY-2, and PGY-3 residents were randomly separated into four groups during a two-hour block reserved for resident didactics. All residents in attendance were enrolled in the simulation training intervention, which took place at the Rector Clinical Skills and Simulation Center (RCSSC) at Thomas Jefferson University.

The simulation case (Appendix Figures 9-16) was developed after review of classic pediatric sedation cases commonly performed in the ED. Four resident groups were created. There were three groups of eight residents of mixed PGY level. Due to several resident absences (i.e., secondary to illness, completion of a shift the evening prior, and/or lateness), an additional group of three residents of mixed PGY level was created. Two groups underwent a simulation without the checklist, and the remaining two groups underwent the simulation with the use of the checklist.

Each group of residents was given the same pediatric case requiring sedation for management, using a SimBaby (Laerdal) model, in a standardized room in the University Simulation Center. The case prompted them to use ketamine for sedation and then encounter the known complication of laryngospasm. As previously described, two of the four groups were randomly chosen to receive the pediatric sedation checklist immediately prior to performing the sedation, with case instructions to utilize it as the new hospital sedation protocol. The remaining two groups were not given the sedation checklist; these groups served as a control for resident performance.

Each group was evaluated on its ability to successfully recognize and perform the critical actions of the case. The primary outcome was percentage of the list of critical actions can be found in Appendix Figure 12. Immediately following the simulation, residents participated in a structured debriefing, facilitated by an EM faculty member, of the case with a discussion of the critical actions and the learning points. Faculty facilitators were provided with a standardized debriefing format to allow for the identical structured debriefing of each group. Residents were then given a post-examination, identical to the pre-test and survey. Data on clinical improvement, as well as changes in attitudes and comfort with the procedure, were collected.

## Results

A total of 36 pre-intervention surveys were returned (response rate 94.7%). Survey results are listed in Table 1. Twelve residents in each class completed the survey. Collectively, residents scored a mean value of 2.43 out of 5 in terms of their confidence with pediatric sedation for patients ranging from infancy to two years of age. Mean confidence score increased to 3.05 for patients aged 2 to 18 years. Senior residents were more comfortable across all age ranges in sedation (score 4.27) compared to interns (1.46 and 1.92). Less than half of PGY-1 residents had performed a pediatric sedation, while the majority of the PGY-3 class had performed 10 or more. As a whole, EM residents were most comfortable with ketamine for sedation (3.03), while senior residents were more comfortable with all sedation medications when compared to junior learners. Confidence with the use of ETCO_2_ waveform interpretation was similar across all learner groups (range: 3.00-3.82).

**Table 1:** Pre-intervention survey results PGY, post-graduate year

	PGY1	PGY2	PGY3	Aggregate
Confidence in sedation (infants to 2-year-olds) (1-5)	1.46±0.66	2.54±0.97	4.27±1.13	2.43±1.21
Confidence in sedation (2- to 18-year-olds) (1-5)	1.92±0.86	3.15±0.99	4.27±0.47	3.05±1.25
Confidence in intubation (pediatrics) (1-5)	1.85±0.80	2.62±1.04	3.09±1.30	2.49±1.15
Comfort with ketamine (pediatrics) (1-5)	2.80±1.33	3.00±1.08	3.82±0.87	3.03±1.24
Comfort with propofol (pediatrics) (1-5)	1.85±0.90	2.54±1.13	3.64±0.92	2.62±1.21
Comfort with midazolam (pediatrics) (1-5)	2.00±0.82	2.69±1.03	3.45±1.13	2.68±1.13
Confidence in reading ETCO_2_ waveform (1-4)	3.00±1.08	3.08±0.95	3.82±1.08	3.27±1.07
Challenges in performing sedation)	Lack of experience (5), medication dosing (5), parents (3)	Lack of experience (3), medication dosing (7), parents (3)	Lack of experience (3), medication dosing (3), parents (3)	Lack of experience (11), medication dosing (15), parents (9)
How many sedations were previously performed?	None (6), 1-5 (6), 5-10 (1)	None (2), 1-5 (7), 5-10 (4)	1-5 (2), 5-10 (2), 10+ (7)	None (8), 1-5 (15), 5-10 (7), 10+ (7)

A total of 27 residents underwent the simulation intervention. The characteristics of the four groups can be found in Table 2. In total, 11 residents served as controls, and 17 residents received the checklist intervention.

**Table 2:** Learner demographics and critical action per group PGY, post-graduate year

Group number	1 (no checklist)	2 (no checklist)	3 (checklist)	4 (Checklist)
Number of residents	3	8	8	8
PGY level of resident (year)	1, 1, 2	1, 1, 2, 2, 2, 2, 2, 3	1, 1, 1, 1, 1, 2, 2, 3	1, 2, 3, 3, 3, 3, 3, 3
Total number of correct critical actions performed	11	10	14	13

A comparison of the critical actions achieved between groups can also be found in Table 2. Residents supplied with the checklist obtained at least three more actions than the lowest-scoring control group. The most frequently missed critical action in both control and intervention groups was termination of the procedure once respiratory distress was detected. In one control group, the critical actions of performing a complete evaluation of the airway and applying appropriate monitors were omitted. The pre-sedation evaluation and preparation actions were never missed in the intervention groups.

The survey results for the residents post-intervention, divided by intervention group, can be found in Table 3. Statistical comparisons were performed using Student's t-test. As a whole, across all survey questions, excluding comfort with propofol and midazolam for sedation, the control group reported higher confidence levels. Senior residents were more comfortable across all age ranges for sedation (3.71-4.00 in the intervention arm, 5.00 in control) than junior residents (2.83-3.00 in both groups). Ketamine was the medication most residents were familiar with (3.91 control, 3.75 intervention). Residents post-intervention across all PGY levels, with the exception of reading an ETCO_2_ waveform, showed increased confidence compared to those surveyed prior to the simulation.

**Table 3:** Comparison of post-simulation survey results *Denotes significant difference defined as P<0.05 when compared to pre-intervention ETCO_2_, end-tidal carbon dioxide; PGY, post-graduate year

	PGY 1	PGY2	PGY 3	Aggregate
Post-intervention survey (control)				
Confidence in sedation (infant)	2.83±0.96*	3.17±0.75	5.00±0.00	3.18+0.98*
Confidence in sedation (child)	3.50±0.82*	3.67±0.52	5.00±0.00	3.75±0.82
Confidence in pediatric intubation	2.75±0.96	3.83±0.75*	3.00±0.00	3.37±0.92*
Comfort with ketamine	3.20±1.26	4.17±0.75*	5.00±0.00	3.91±1.04*
Comfort with propofol	2.75±0.96	3.67±1.03*	4.00±0.00	3.36±1.03
Comfort with midazolam	2.75±1.26	3.17±0.75	4.00±0.00	3.09±0.94
Confidence in reading ETCO_2_ waveform	2.75±0.96	3.17±0.41	3.00±0.00	3.09±0.70*
Post-intervention survey (intervention)				
Confidence in sedation (infant)	2.83±1.17*	4.00±0*	3.71±0.76	3.44+0.96*
Confidence in sedation (child)	3±1.26*	4.00±0	4.00±1.00	3.63+1.09
Confidence in pediatric intubation	2.33±0.82	3.00±0	3.57±0.79	3.00+0.89
Comfort with Ketamine	3.5±1.38	3.33±0.58	4.14±1.07	3.75+1.13*
Comfort with Propofol	3.5±1.17*	3.33±0.58	4.14±1.11	3.75+1.20
Comfort with Midazolam	2.83±1.05	3.67±1.00	4.29±1.07	3.63+1.11*
Confidence in reading ETCO_2_ waveform	2.5±0.52	3.00±0.00	3.86±0.69	3.19+0.57*

An analysis of pre- and post-intervention examination results can be found in Table 4; analysis was conducted using Student's t-test. Residents collectively scored an average of 63% on the pre-test. The most commonly missed questions involved pediatric endotracheal tube size, depth of insertion for intubation, and performing maneuvers (18/36 correct). Recognition of hypopnea on an ETCO_2 _tracing was marked correct by the majority of learners (32/36).

**Table 4:** Pre- and post-intervention examination comparison *No significant difference found between pre-intervention and either control or intervention group defined as P<0.05 **No significant difference found between control and intervention groups defined as P<0.05 PGY, post-graduate year

	Pre-intervention examination	Post-intervention examination (without checklist)	Post-intervention examination (with checklist)
PGY level	Total score	Total score	Total score
1	6.58±2.43*	7.5±1.91**	7.67±1.21**
2	6.08±1.62*	7.17±1.33**	7.67±0.58**
3	6.17±2.44*	5.00±0.00**	5.57±2.15**
Aggregate	6.28±2.14*	7.09±1.58**	6.75±1.88**

Residents post-simulation scored higher on the examination than pre-intervention in PGY-1 and PGY-2 levels by at least one point, with the largest increase noted in the PGY-2 group of 1.5 points in the group provided with the safety checklist. PGY-3 residents scored less than the pre-intervention arm. When comparing the post-simulation groups, PGY-2 and PGY-3 residents who were provided the checklist scored at least half a point more than their counterparts in the control. The most frequently missed question included pre-airway evaluation (6/11 control, 8/16 intervention); however, the learners improved on recognizing appropriate pediatric endotracheal tube and depth of insertion.

## Discussion

The main purpose of the study was to enhance patient safety by introducing a procedural checklist for the practice of pediatric sedations. Based on evaluation of critical actions, the groups with the checklist obtained several more critical actions, and pre-sedation evaluation and preparation actions were not missed. The second purpose of the study was to assess for change in attitudes and confidence in performing pediatric sedation among residents and to assess for knowledge improvement. Residents surveyed in the post-simulation groups scored slightly higher in confidence than the pre-simulation survey, with the control group interestingly scoring the highest in confidence, despite performing worse on the simulation. This may imply that simulation alone without stimuli to focus on critical actions may lead to a sense of false confidence.

The survey data revealed improvement in scores post-simulation for PGY-1 and PGY-2 residents, with the largest increase in examination scores observed in the intervention group that was provided with the checklist. This may suggest that a checklist alone is not sufficient to improve confidence in trainees and that simulation practice followed by a debriefing is necessary to improve knowledge and attitudes. This is confirmation of the importance of simulation and debriefing in rare procedures, such as pediatric simulation [13,14].

Several observations were made during the simulations. While groups were not randomized, one group had a majority of senior residents (i.e., PGY-3); despite being provided with the checklist, this group did not score the highest number of critical actions (13 vs. 14). Furthermore, senior residents did not attain the highest scores on the post-intervention examination. In the intervention arm, PGY-2 residents scored higher than the combined PGY-3 residents. During the sedation simulation, senior residents allowed junior residents to run the simulation and only intervened when a perceived critical action was about to be omitted. The group with the majority of PGY-1 residents, however, scored the highest number of critical actions out of all groups provided with the checklist. This may suggest that residents earlier in their training are more reliant on prompts and scaffolded instruction than their more senior counterparts. This also comports with established literature, which reveals that while all levels of learners can benefit from checklists, novices benefit the most [15,16].

There are several additional reasons for this discrepancy. The study took place in the second half of the academic year. It is likely that PGY-3 residents were not fully engaged in the simulation, the survey, or even the examination. To this point, one PGY-3 resident consistently marked “5” for all survey answers, and another chose to mark all the same letter answer choice on each examination question. In addition, junior residents may have appreciated the opportunity to practice pediatric sedation during the simulations and were, therefore, more eager to participate and meet more of critical actions. Future studies should evaluate and place emphasis on learner engagement to better standardize evaluation.

Though the groups were not randomly assigned, a majority of the junior learners present (PGY-1 and PGY-2 residents) were coincidentally distributed in roughly even numbers across control and intervention groups. Making the sedation checklist available improved the number of critical actions achieved, suggesting that having a procedural checklist available improves and reinforces safe practice for procedural sedation. While the sample of residents in our study is small, more investigation is needed to elucidate if this improvement in critical actions is sustained in subsequent samples.

In the post-simulation survey, learners in the checklist group interestingly scored a lower aggregate confidence level than those in the control (i.e., without the checklist). Though this difference was small, one reason for this discrepancy may be that the checklist prompted areas and/or steps that residents may not have originally known or taken, highlighting potential knowledge gaps. Further study is needed with qualitative analysis to better comprehend this observed phenomenon.

Our simulations covered several ACGME EM milestones, including but not limited to Diagnosis (PC4), Pharmacotherapy (PC5), Airway Management (PC10), Anesthesia and Acute Pain Management (PC11), Vascular Access (PC14), Medical Knowledge (MK), Patient Safety (SBP1), Patient-Centered Communication (ICS1), and Team Management (ICS2). As a result, our training program has the potential to be developed as an in situ, just-in-time training (JITT) module for pediatric procedural sedation for EM residents that can take place in the clinical environment, just prior to application. Simulation training, specifically JITT - where training takes place on demand immediately before the procedure in the clinical environment or as an immediate stimulus after clinical theory review - presents a ripe opportunity to review procedural steps, support the development of a shared mental model among providers, allow for deliberate practice of procedural skills under the supervision of a senior resident or faculty member for formative feedback, and support the development of muscle memory for procedural skills. Future work should be dedicated to this implementation.

There are several limitations to this study. First, the study was limited to a single, three-year EM residency in an urban environment that does not have a dedicated pediatric ED. This may limit the generalizability of the results. Similarly, the training was only given once to a single cohort of residents, and not all residents completed either the pre-intervention survey or the simulation session. Further training sessions in sequential years would improve the power of the results and may reveal that inter-class differences represent different skill sets between individuals within classes as compared to just level of experience. The simulation proctors were not blinded to the intervention, perhaps confounding the data collection. While this may have resulted in unconscious bias, the critical actions were clearly delineated and only assessed when performed without prompting. Our third-year residents were also not evenly distributed, which could have impacted the results, though it is important to note that the group with the most interns and the checklist did better than the group with mostly senior residents. Lastly, this was a single educational session and did not evaluate clinical outcomes. Future studies should assess the value of these sessions on patient outcomes and long-term retention of knowledge.

## Conclusions

Pediatric sedation is a low-frequency procedure for many adult EDs, therefore requiring frequent review of rescue techniques and medication considerations. Our study represents our efforts to standardize training and practice of pediatric sedations in the ED using quality improvement methodologies. Preliminary data suggests that a safety checklist, combined with the opportunity for practice in a simulation center, may reinforce pre-procedural techniques and steps that would and allow for review of critical actions should an emergent situation develop during a sedation. Future studies should attempt to evaluate longitudinal improvement in resident performance after repeated training sessions with such a checklist.

## Figures and Tables

**Figure 1:**
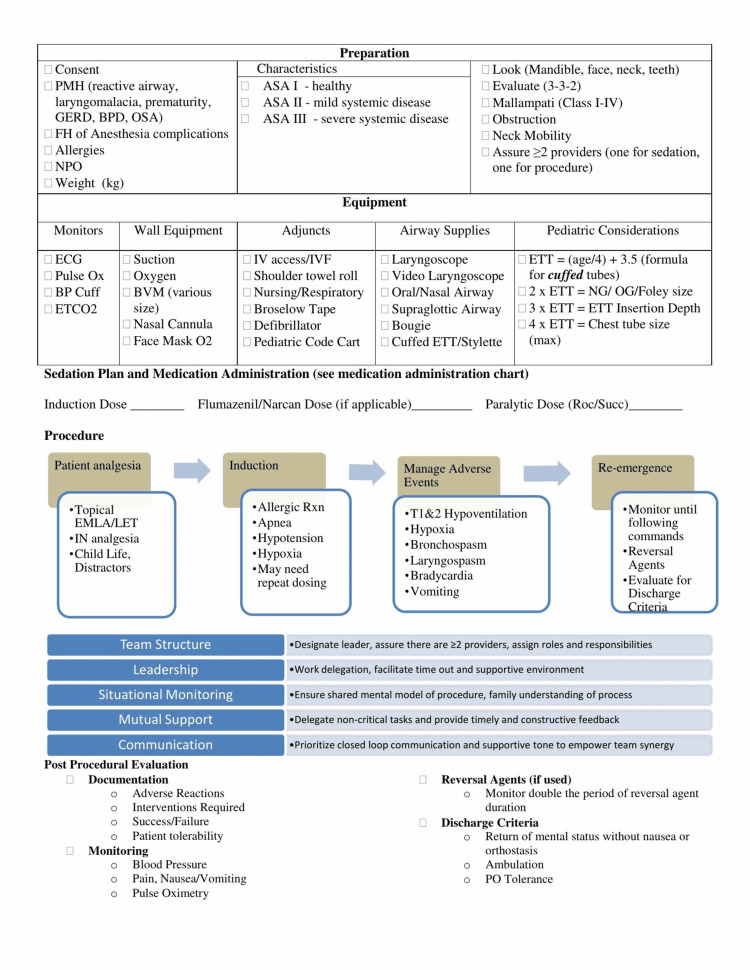
Procedural sedation checklist

**Figure 2:**
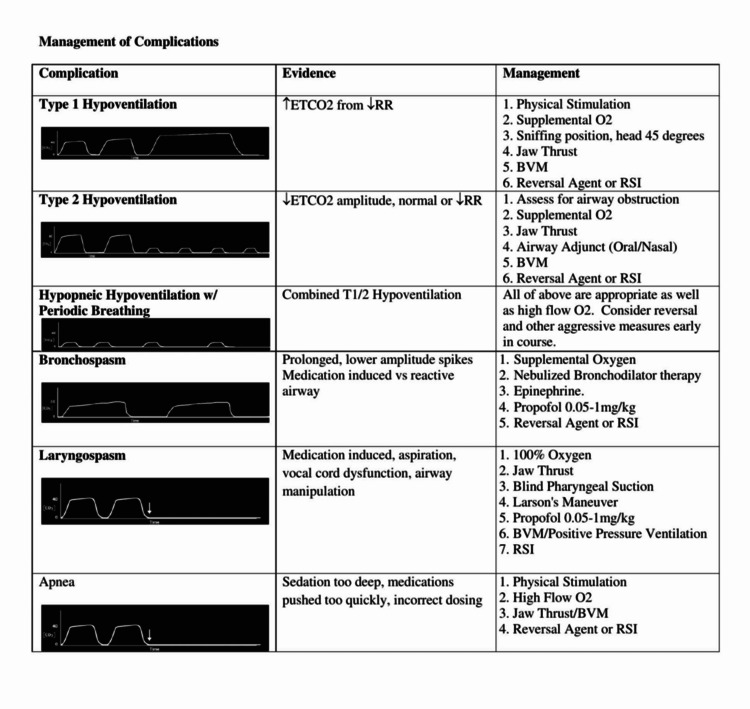
Capnography waveforms and management

**Figure 3:**
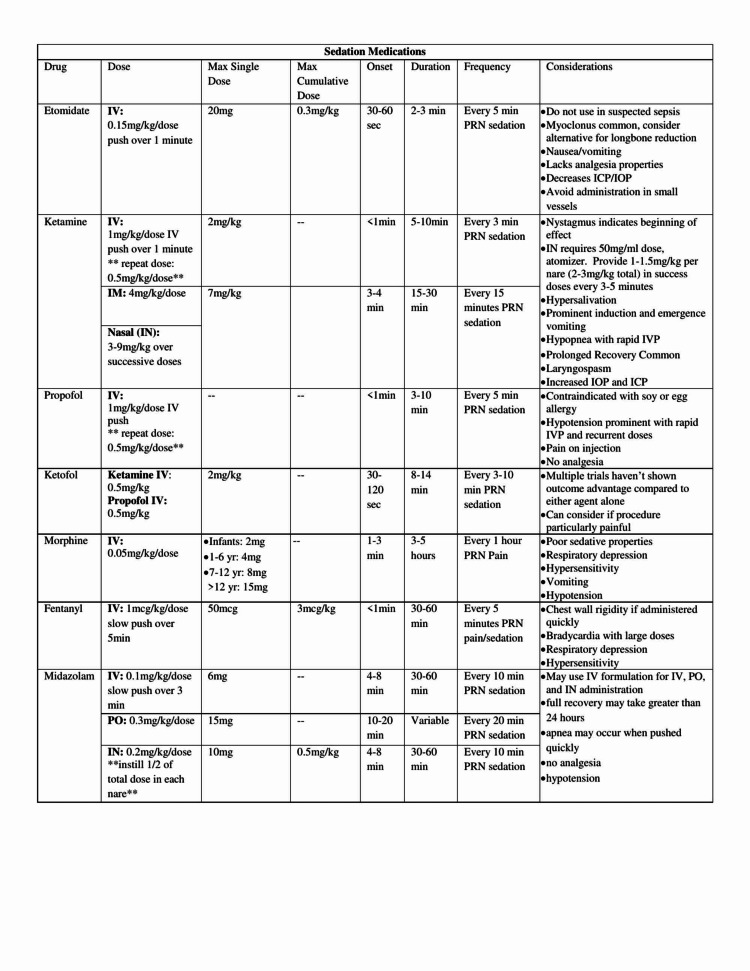
Sedation medications reference

**Figure 4:**
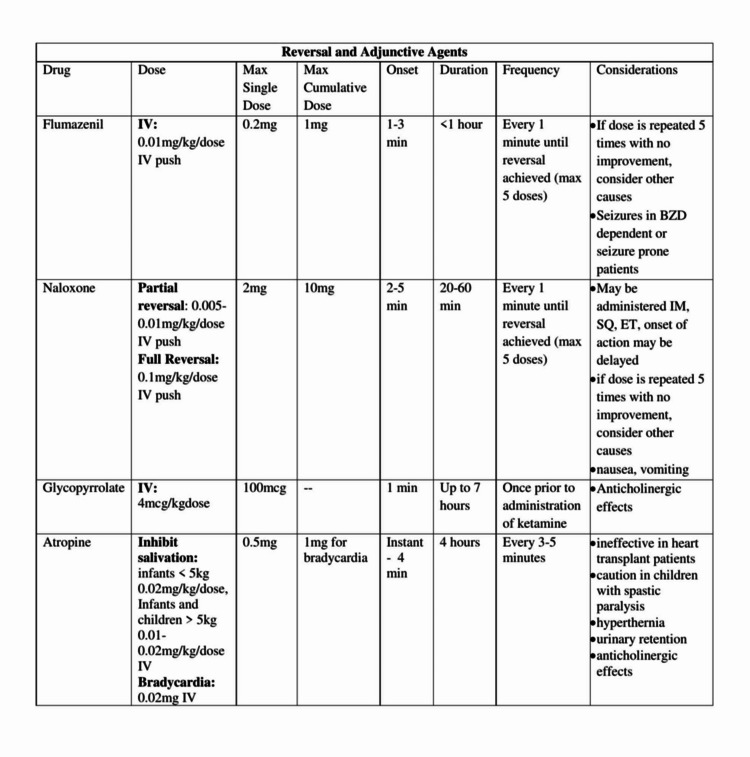
Reversal medications reference

**Figure 5:**
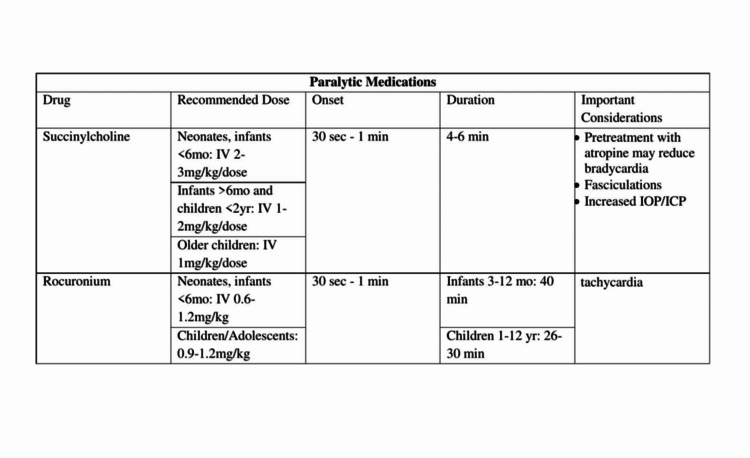
Paralytic medications reference

**Figure 6:**
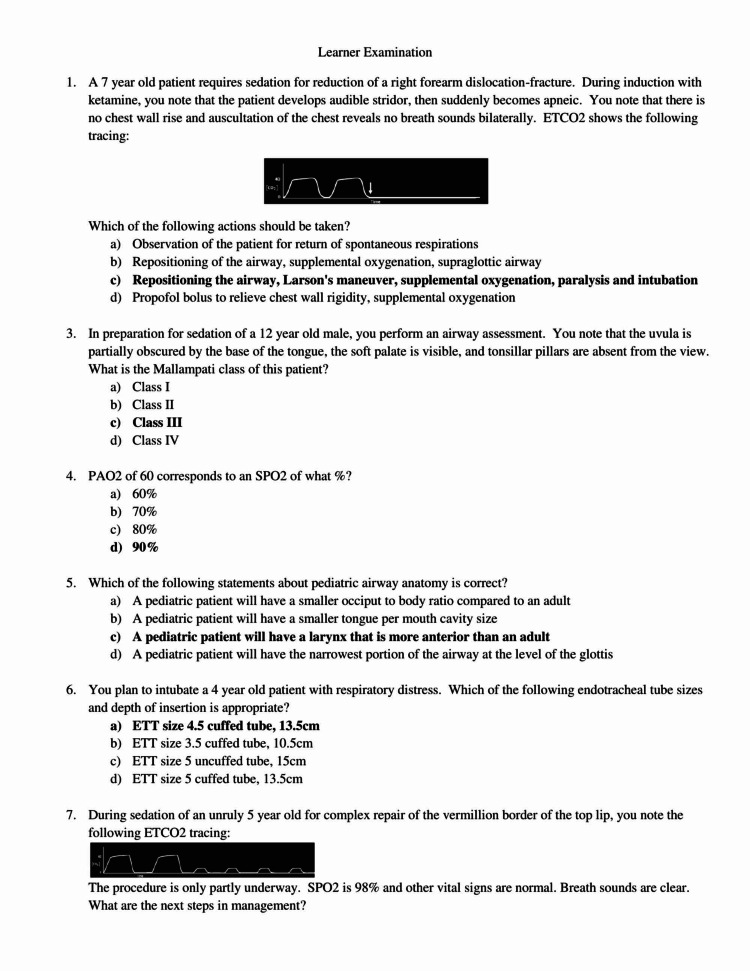
Learner assessment page 1

**Figure 7:**
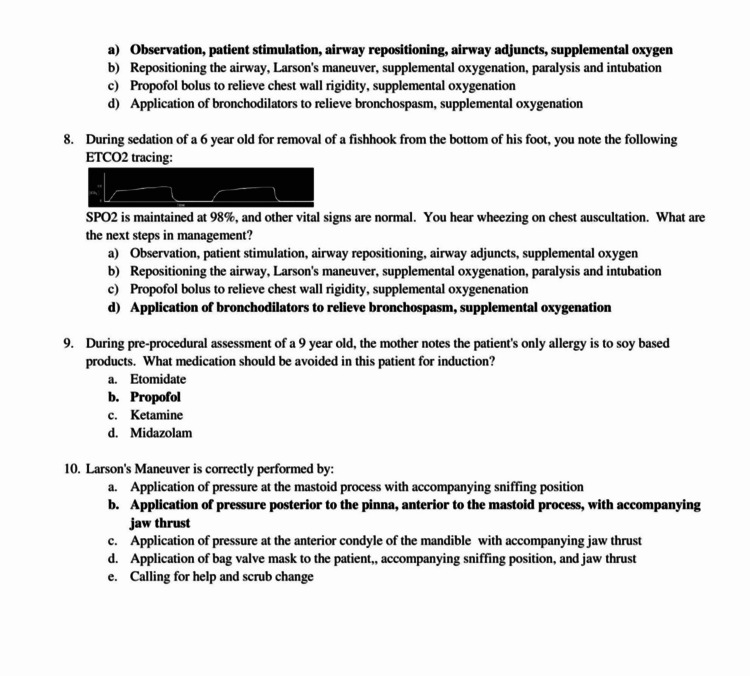
Learner assessment page 2

**Figure 8:**
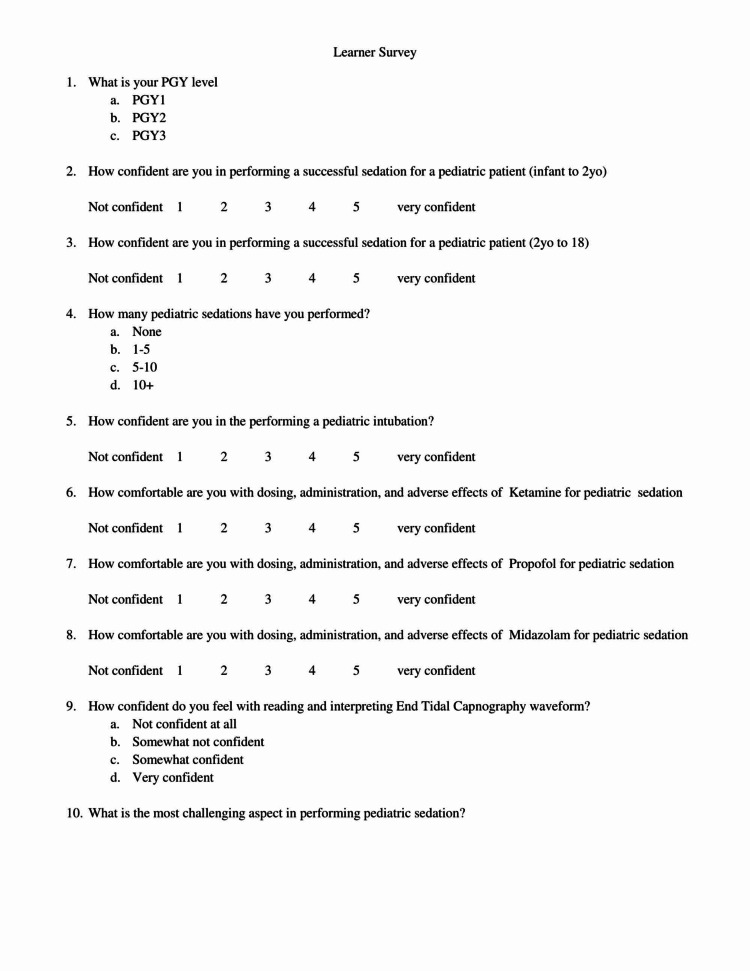
Learner pre- and post-intervention survey

**Figure 9:**
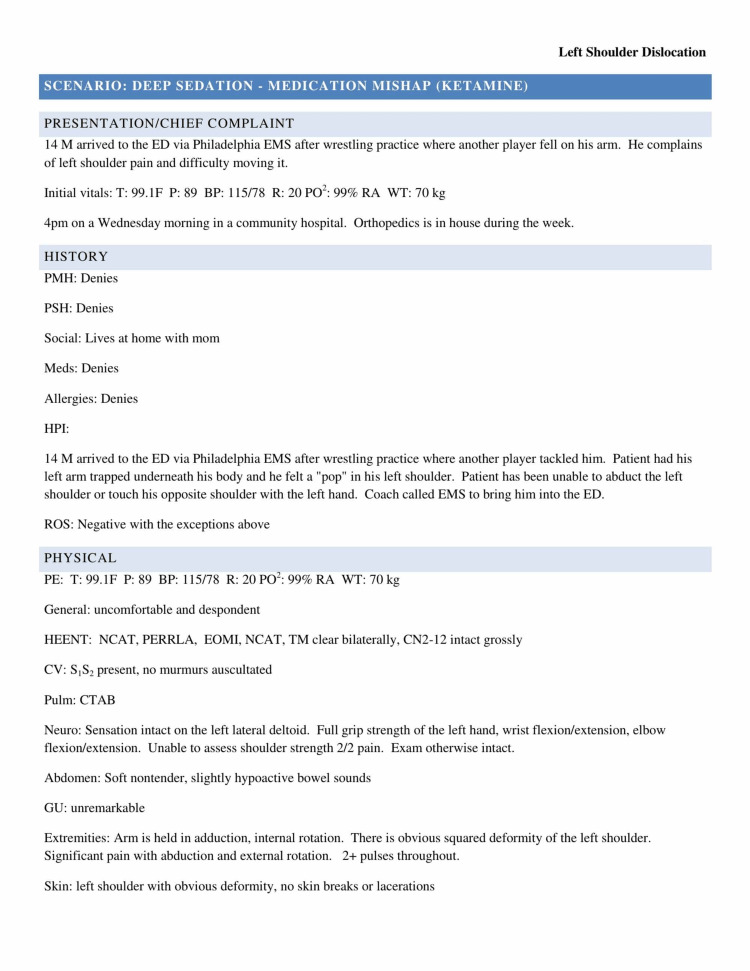
Simulation case page 1

**Figure 10:**
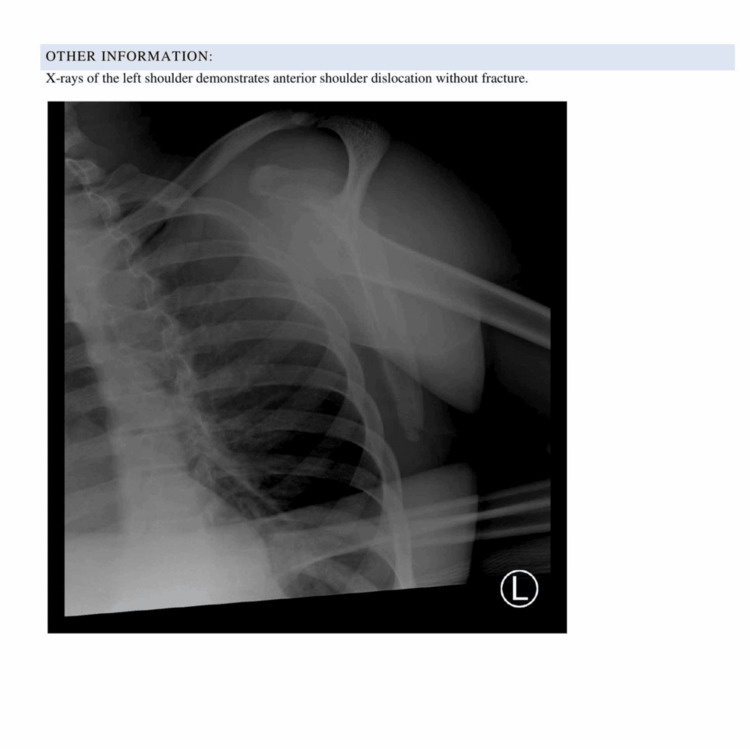
Simulation case page 2

**Figure 11:**
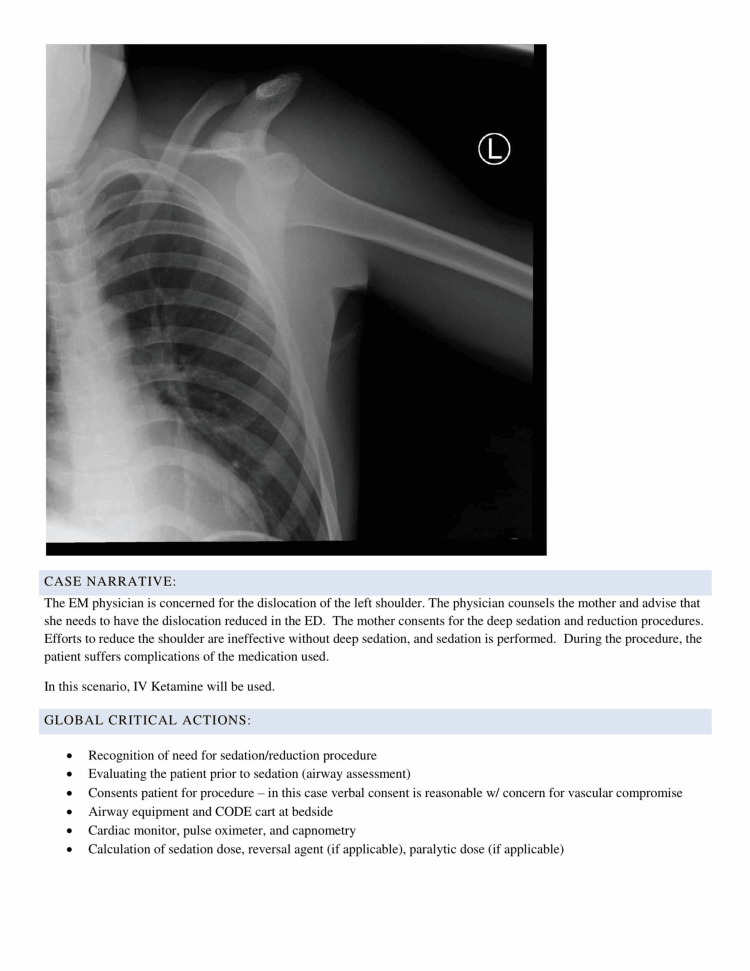
Simulation case page 3

**Figure 12:**
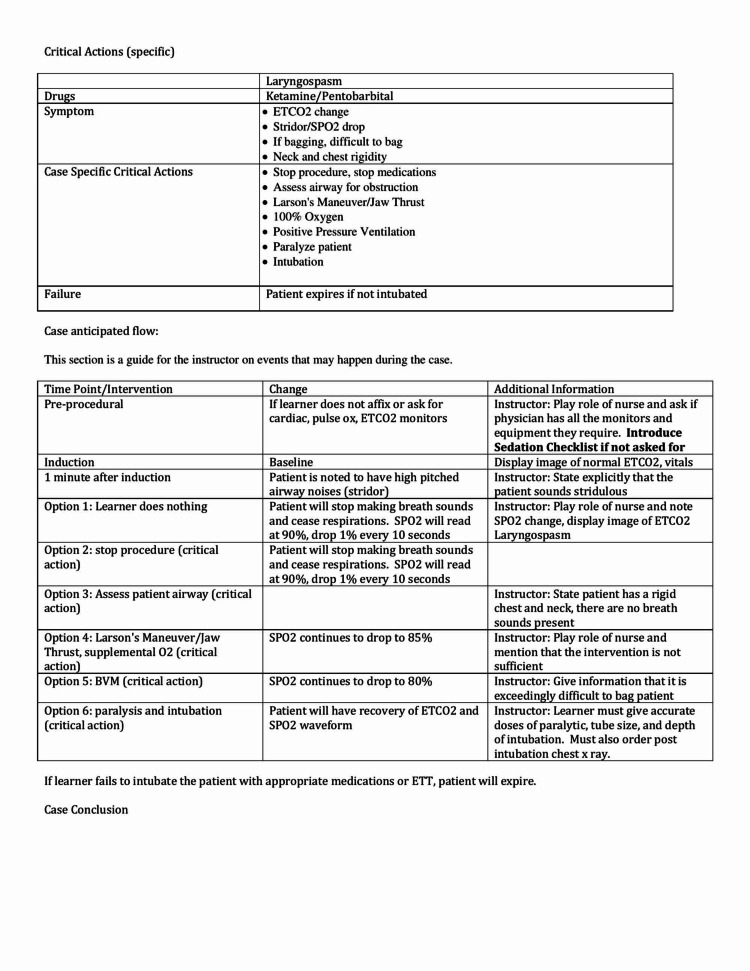
Simulation case page 4

**Figure 13:**
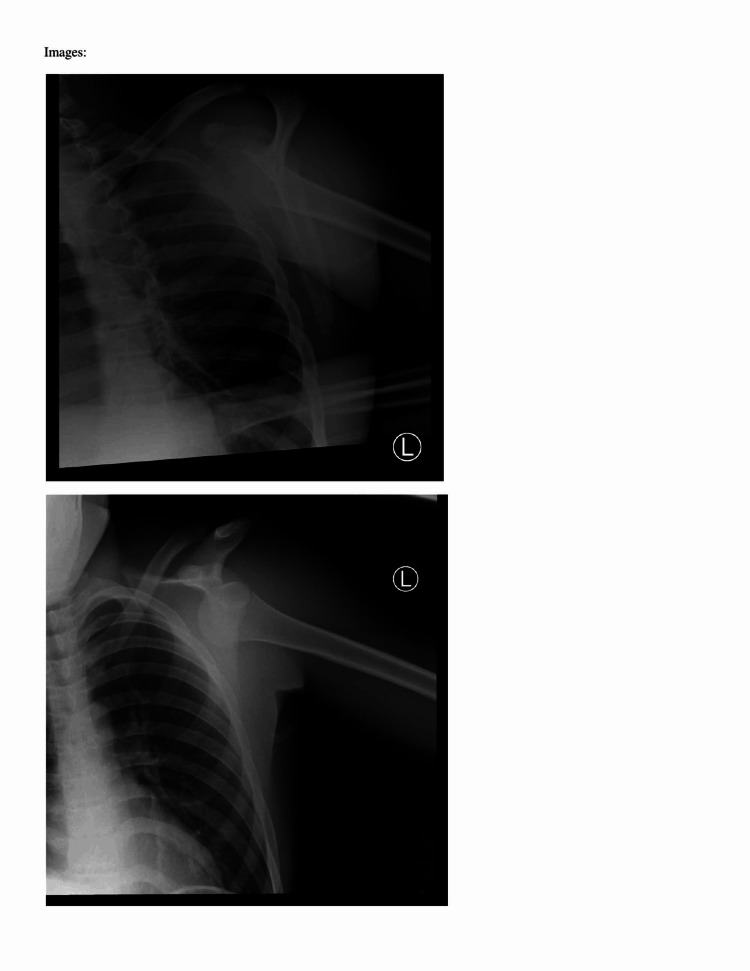
Simulation case page 5

**Figure 14:**
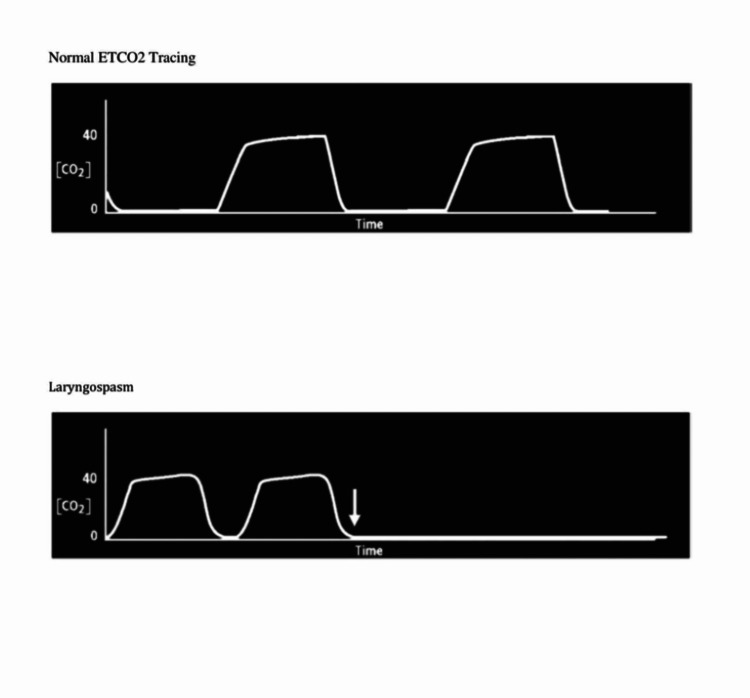
Simulation case page 6

**Figure 15:**
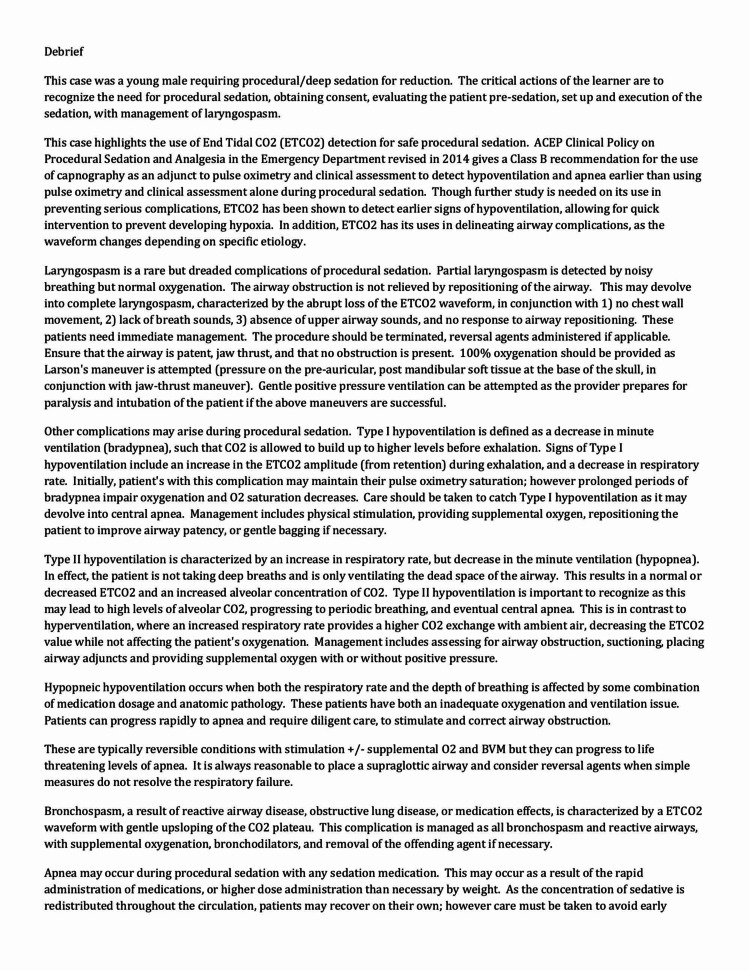
Simulation case page 7

**Figure 16:**
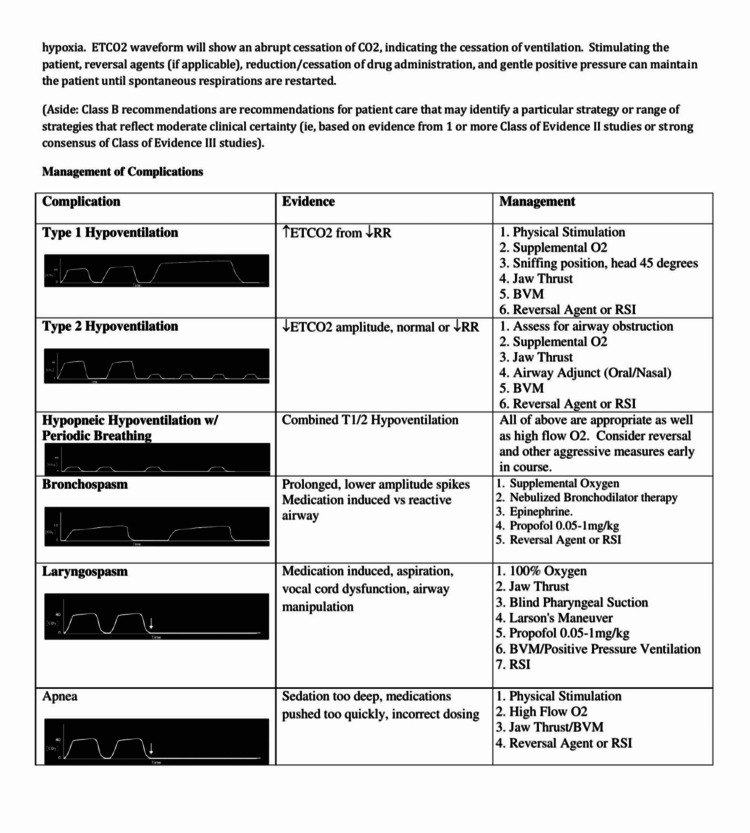
Simulation case page 8
